# Smurf2 enhances ubiquitin-mediated degradation of CASC3 and attenuates leukemia progression

**DOI:** 10.1016/j.isci.2025.113411

**Published:** 2025-08-21

**Authors:** Ronghao Zeng, Jing Liu, Fen Lu, Ming Hong, Ting Lan, Baijian Chen, Yunping Pu, Yuwei Tan, Peter Wang, Juan Wang, Weijia Wang

**Affiliations:** 1 Department of Advanced Diagnostic and Clinical Medicine, Zhongshan City People’s Hospital, Zhangshan, Guangdong 528403, China; 2 Department of Clinical and Experimental Medicine, Zhongshan City People’s Hospital, Zhangshan, Guangdong 528403, China; 3 Department of Medicine, Beijing Zhongwei Research Center, Biological and Translational Medicine, Beijing 100161, China

**Keywords:** Biochemistry, Cell biology, Cancer

## Abstract

Leukemia is a prevalent cancer worldwide with a poor prognosis, underscoring the need to understand the molecular mechanisms driving its development and progression. Smurf2 has been implicated in the development of numerous cancer types; however, its specific role in leukemia remains elusive. In this study, we investigated the function of Smurf2 in leukemia progression. Our findings demonstrate that upregulation of Smurf2 reduces viability of leukemia cells and induces apoptosis. Moreover, we identified CASC3 as a substrate of Smurf2 in leukemia. Smurf2 interacts with CASC3 and regulates its ubiquitination and degradation. Notably, Smurf2-mediated degradation of CASC3 depends on its 137–283 domain and lysine residue K254. Strikingly, the downregulation of Smurf2 promotes cell viability through CASC3, while overexpression of Smurf2 retards tumor growth in mouse models. Collectively, our results suggest that the Smurf2/CASC3 axis may serve as a potential therapeutic target for the treatment of leukemia.

## Highlights

• Smurf2 reduces viability of leukemia cells and induces apoptosis
• CASC3 is a substrate of Smurf2 in leukemia
• Smurf2-mediated degradation of CASC3 depends on K254
• Smurf2/CASC3 axis is a potential therapeutic target for leukemia

## Graphical abstract

**Figure:**
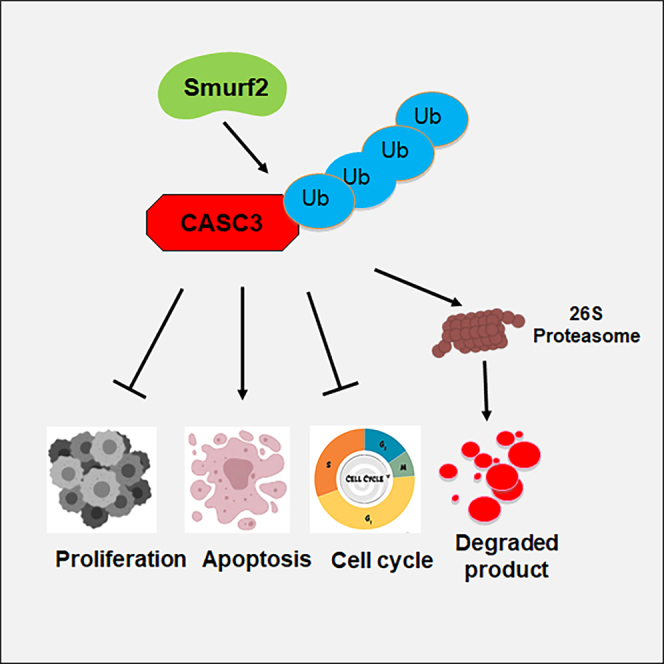

## Introduction

Leukemia ranks as the 13th most commonly diagnosed cancer and the 10th leading cause of cancer-related deaths globally.^1^ In the United States, an estimated 62,770 new cases and 23,670 deaths from leukemia are projected in 2024.^2^ Leukemia comprises various subtypes, including acute lymphoblastic leukemia (ALL), acute myeloid leukemia (AML), chronic lymphocytic leukemia, and chronic myeloid leukemia (CML).^3^ Targeted therapies and immunotherapies have shown promising efficacy in treating subgroups of leukemia patients. The advent of chimeric antigen receptor (CAR) T cell therapy represents a significant breakthrough in leukemia management.^4^^,^^5^ Furthermore, the introduction of tyrosine kinase inhibitors (TKIs), such as imatinib, have enabled most CML patients to achieve near-normal life expectancy.^6^ However, drug resistance and the risk of progressing to acute leukemia remain concerns for 5%–10% of CML patients.^7^ To pave the way for more effective and personalized treatment strategies, a deeper understanding of the molecular and immunological underpinnings of leukemia is essential.

Post-translational modifications play critical roles in regulating various cellular processes by altering protein function, stability, and localization.^8^^,^^9^^,^^10^ These modifications include phosphorylation, acetylation, ubiquitination, methylation, and glycosylation.^11^^,^^12^ Ubiquitination, in particular, involves the covalent attachment of ubiquitin to a lysine residue on a target protein,^13^^,^^14^ orchestrated by a cascade of enzyme activities: E1 (ubiquitin-activating enzyme), E2 (ubiquitin-conjugating enzyme), and E3 (ubiquitin ligase).^15^ Smurf2 (Sma and Mad ubiquitination regulatory factor 2), an E3 ligase belonging to the neuronally expressed developmentally downregulated 4 family of proteins,^16^^,^^17^ has been implicated in tumorigenesis across various cancer types. For example, Smurf2 has been shown to target ring finger protein 11 (RNF11), Smurf1, and connector enhancer of kinase suppressor of Ras 2 (CNKSR2) for ubiquitination and degradation in breast cancer.^18^^,^^19^ In colorectal cancer, Smurf2 promotes the ubiquitination and degradation of carbohydrate response element-binding protein (chREBP), thereby reducing aerobic glycolysis and inhibiting cell proliferation.^20^ Additionally, Smurf2 mediates the degradation of SIRT1, leading to suppressed cell proliferation and tumorigenesis in colorectal cancer.^21^ In lung cancer, Smurf2 enhances epidermal growth factor receptor (EGFR) stability, contributing to resistance against TKI.^22^ Similarly, Smurf2 stabilizes EGFR in lung cancer and cervical cancer, indicating that Smurf2 could be an oncogene in lung and cervical cancers.^23^ Smurf2 promotes membrane displacement of GTPase-activating protein 17 (GAP17) isoform 1 to enhance oncogenic synergy between mutant p53 and Kirsten rat sarcoma viral oncogene homolog (KRAS).^24^ Smurf2 promotes ribosomal protein L35A (RPL35A) polyubiquitination and degradation, leading to inhibition of proliferation and cell-cycle progression in breast cancer.^25^ Although Smurf2 has been extensively studied in various cancers, its role in leukemia remains to be elucidated.

CASC3 (cancer susceptibility candidate 3), also known as MLN51 (metastatic lymph node 51), is a core component of the exon junction complex (EJC) and plays a crucial role in RNA processing, particularly in mRNA splicing.^26^ High expression of MLN51 has been observed in malignant epithelial cells of human breast carcinomas.^27^ MLN51 has been shown to associate with the EJC through its speckle localizer and RNA-binding modules.^28^ Overexpression of MLN51 in HER2-positive human breast cancer cells is associated with a reduction in P-body numbers, linking P-body disassembly, mRNA deregulation, and cancer progression.^29^ Increased MLN51 copy numbers have also been observed in gastric cancer.^30^ However, the role of CASC3 (MLN51) in leukemia development and progression remains poorly understood. This study aims to elucidate the function and underlying mechanisms of CASC3 in leukemia. In the current study, we report that Smurf2 enhances CASC3 ubiquitination and degradation, thereby attenuating leukemia progression.

## Results

### Downregulation of Smurf2 facilitates viability of leukemia cells

**Figure 1:**
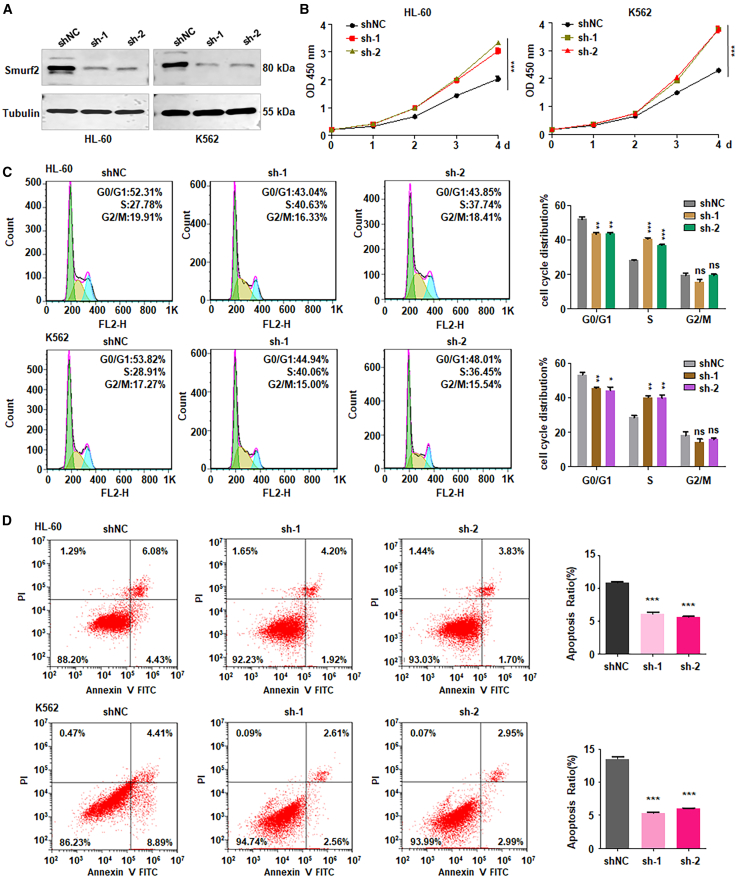
Downregulation of Smurf2 facilitates viability of leukemia cells (A) Western blotting analysis of Smurf2 expression in HL-60 and K562 cells following shSmurf2 infection. (B) CCK-8 assay assessing cell viability in HL-60 and K562 cells post-shSmurf2 infection. (C) Cell-cycle analysis in HL-60 and K562 cells post-shSmurf2 infection. Right panel: Quantitative data for the left panel. (D) Cell apoptotic death analysis in HL-60 and K562 cells following shSmurf2 infection. Right panel: Quantitative data for the left panel. Data are representative of three independent experiments. The data are presented as the mean ± SD. *n* = 3. ∗∗*p* < 0.01; ∗∗∗*p* < 0.001; ns, no significance.

Smurf2 has been identified as a tumor suppressor gene and is associated with prognosis in a variety of human cancers.^17^ However, the role of Smurf2 in leukemia development remains unclear. Using two online databases (https://kmplot.com and https://oncolnc.org), we observed that AML patients with high expression of Smurf2 displayed better survival outcomes compared with those with low expression of Smurf2 (Figure S1). To determine the function of Smurf2 in leukemia, shRNA Smurf2 (shSmurf2) plasmids were infected into HL-60 and K562 cells. To detect the efficacy of shSmurf2 infection in leukemia cells, western blotting analysis was used to determine the expression of Smurf2 in HL-60 and K562 cells after shSmurf2 transfection. The data from western blotting showed that both shSmurf2-1 and shSmurf2-2 plasmids remarkedly reduced the expression of Smurf2 in HL-60 and K562 cells (Figures 1A and S2A). Moreover, the cell counting kit-8 (CCK-8) assay was performed in HL-60 and K562 cells after shSmurf2 infection. We observed that shSmurf2 significantly facilitated cell viability in both HL-60 and K562 cells at 72 and 96 h post-transfection (Figure 1B).

### Downregulation of Smurf2 influences cell cycle and apoptosis

To determine whether Smurf2 influences cell cycle in leukemia cells, we explored cell-cycle distribution in HL-60 and K562 cells following shSmurf2 infection. The data demonstrated that Smurf2 knockdown increased the proportion of cells in the S phase compared to the shRNA negative control (shNC) group in both HL-60 and K562 cells (Figure 1C). Given that apoptosis contributes to suppressing cell viability, we also measured apoptotic cell death in Smurf2-depleted HL-60 and K562 cells. The results indicated that Smurf2 knockdown reduced apoptosis, including early and late apoptosis, in both HL-60 and K562 cells (Figure 1D). Moreover, we observed that Smurf2 knockdown increased the expression of Bcl-2 in leukemia cells (Figures S2B and S2C). Collectively, these findings suggest that downregulation of Smurf2 facilitated cell proliferation and inhibited apoptosis in leukemia.

### Upregulation of Smurf2 modulates cell viability, cell cycle, and apoptosis

**Figure 2:**
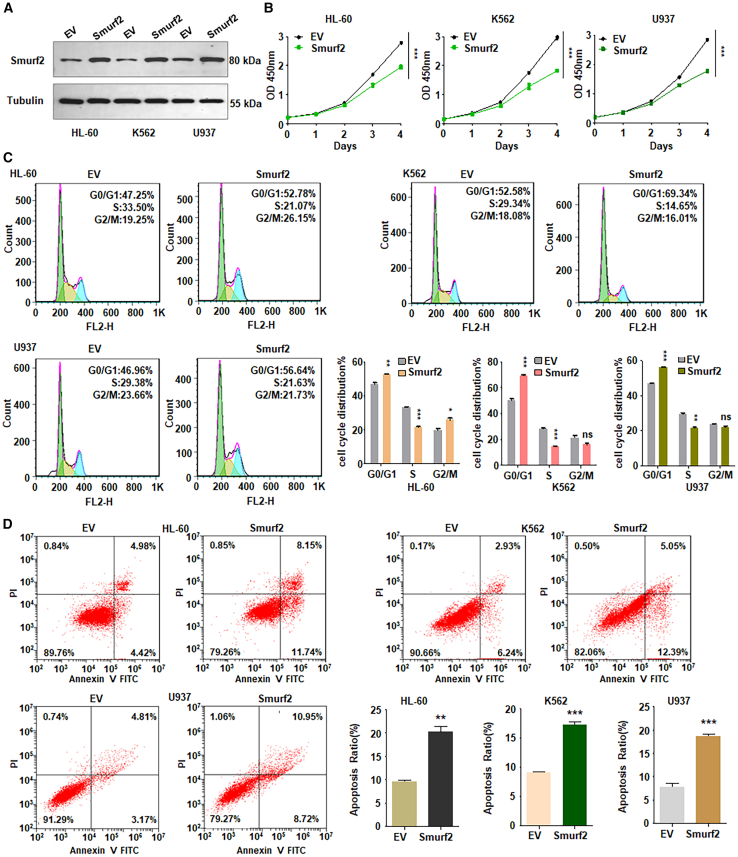
Upregulation of Smurf2 reduces viability of leukemia cells (A) Western blotting analysis of Smurf2 in HL-60, K562, and U937 cells after Smurf2 plasmid transfection. (B) CCK-8 assay assessing cell viability HL-60, K562, and U937 cells following Smurf2 plasmid transfection. (C) Cell-cycle analysis in HL-60, K562, and U937 cells post-Smurf2 plasmid transfection. Quantitative data for cell-cycle analysis are illustrated. (D) Cell apoptotic death analysis in HL-60, K562, and U937 cells post-Smurf2 plasmid transfection. Quantitative data for apoptosis analysis are illustrated. Data are representative of three independent experiments. The data are presented as the mean ± SD. *n* = 3. ∗*p* < 0.05; ∗∗*p* < 0.01; ∗∗∗*p* < 0.001; ns, no significance.

To further validate the role of Smurf2 in regulating viability of leukemia cells, we overexpressed Smurf2 in HL-60, K562, and U937 cells. Western blotting analysis showed a marked increase in Smurf2 levels following plasmid transfection compared with empty vector controls (Figures 2A and S2D). CCK-8 assays demonstrated that overexpression of Smurf2 decreased cell viability across all three leukemia cell lines (Figure 2B). Moreover, cell-cycle analysis indicated that increased Smurf2 expression triggered G0/G1 phase arrest in three leukemia cell lines (Figure 2C). For example, in K562 cells, overexpression of Smurf2 induced G0/G1 phase arrest from 52.58% to 69.34% (Figure 2C). Furthermore, upregulation of Smurf2 led to increased apoptosis in all three leukemia cell lines (Figure 2D). For instance, in HL-60 cells, early apoptosis increased from 4.42% to 11.74%, and late apoptosis rose from 4.98% to 8.15% (Figure 2D). Similar trends were observed in U937 cells, where early apoptosis increased from 3.17% to 8.72% and late apoptosis from 4.81% to 10.95% (Figure 2D). Notably, Smurf2 upregulation inhibited the expression of Bcl-2 in leukemia cells (Figures S2B and S2C). Hence, Smurf2 overexpression attenuated cell viability, induced cell-cycle arrest, and stimulated apoptosis in leukemia.

### Smurf2 interacts with CASC3 and regulates its degradation

**Figure 3:**
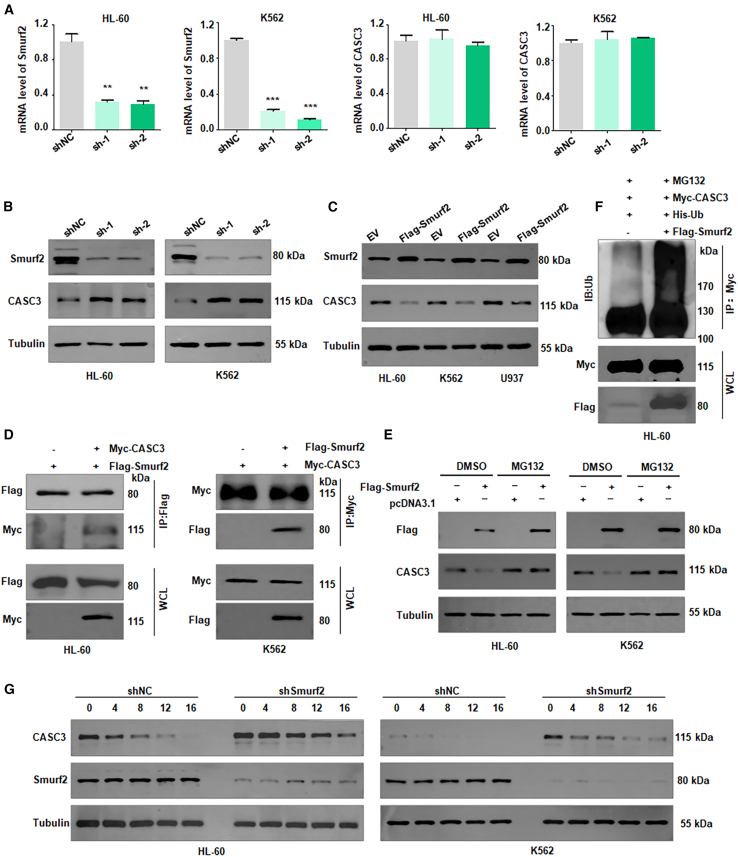
Smurf2 interacts with CASC3 and regulates its degradation (A) Reverse-transcription PCR (RT-PCR) analysis of Smurf2 and CASC3 mRNA levels in HL-60 and K562 cells post-shSmurf2 infection. (B) Western blotting analysis of Smurf2 and CASC3 protein levels in HL-60 and K562 cells post-shSmurf2 infection. (C) Western blotting analysis of Smurf2 and CASC3 protein levels in HL-60, K562, and U937 cells post-Smurf2 plasmid transfection. (D) Immunoprecipitation (IP) assay detecting the interaction between CASC3 and Smurf2 in HL-60 and K562 cells. (E) Western blotting analysis of CASC3 protein levels in HL-60 and K562 cells post-MG132 treatment. (F) Ubiquitination assay in HL-60 cells co-transfected with Myc-CASC3, His-Ub, and FLAG-Smurf2. (G) Cycloheximide chase assay measuring half-life of CASC3 in HL-60 and K562 cells post-shSmurf2 infection. Data are representative of three independent experiments. The data are presented as the mean ± SD. *n* = 3. ∗∗*p* < 0.01; ∗∗∗*p* < 0.001.

To further explore the underlying mechanism of Smurf2-mediated inhibition of cell viability in leukemia, we performed a mass spectrometry analysis in HL-60 cells after Smurf2 plasmid transfection. The results identified CASC3 as a potential Smurf2-interacting protein (Figure S3). To further dissect whether CASC3 is a downstream target of Smurf2, we assessed the expression of CASC3 in leukemia cells after Smurf2 modification. In both HL-60 and K562 cells, shSmurf2 infection decreased the expression of Smurf2 but did not alter CASC3 mRNA levels (Figure 3A). However, CASC3 protein levels were increased in Smurf2-depleted HL-60 and K562 cells (Figures 3B and S4A). Conversely, overexpression of Smurf2 by its plasmid transfection reduced CASC3 protein levels (Figures 3C and S4B). To test whether Smurf2 could bind with CASC3, we performed immunoprecipitation (IP) assays and found that Smurf2 interacts with CASC3 in HL-60 and K562 cells (Figure 3D). To determine whether proteasome-dependent degradation is involved in CASC3 protein regulation, transfected leukemia cells were treated with MG132, a potent cell-permeable proteasome inhibitor. MG132 exposure abolished Smurf2-induced CASC3 degradation in HL-60 and K562 cells (Figure 3E). Importantly, ubiquitination assays showed that upregulation of Smurf2 increased CASC3 ubiquitination in HL-60 cells (Figure 3F). A cycloheximide chase assay was performed to measure half-life of CASC3 in HL-60 and K562 cells after shSmurf2 infection. The data showed that knockdown of Smurf2 prolonged the half-life of CASC3 in both leukemia cell lines (Figures 3G and S4C). These results suggest that Smurf2 mediates the ubiquitination and degradation of CASC3, contributing to the regulation of leukemia cell viability.

### Smurf2-mediated degradation of CASC3 depends on specific domains

**Figure 4:**
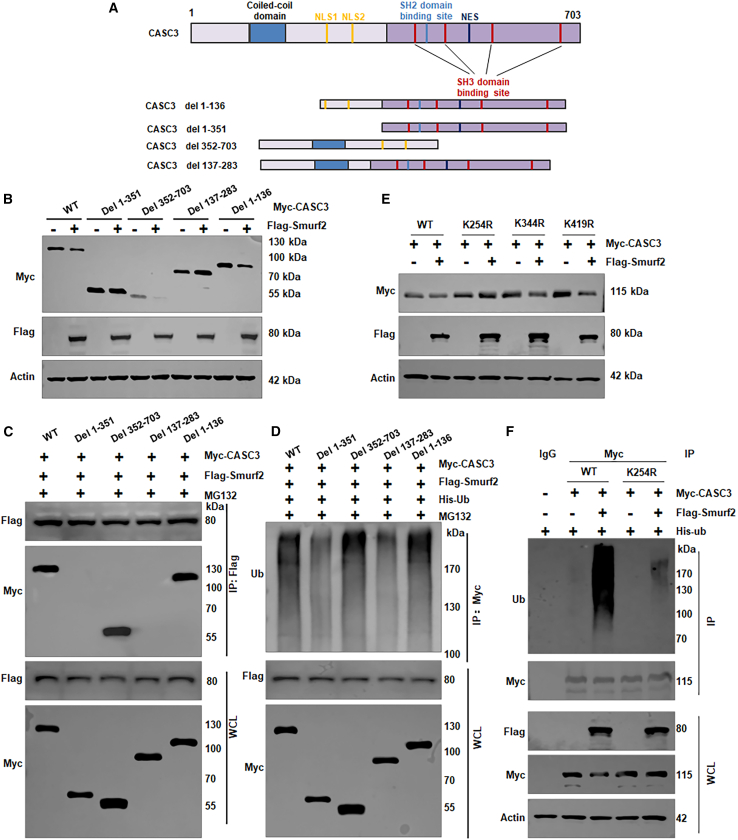
Smurf2-mediated degradation of CASC3 depends on specific domains (A) Schematic representation of CASC3 deletion constructs: deletion of 1–136, 1–351, 137–283, and 352–703. (B) Western blotting analysis of CASC3 expression in HL-60 cells after transfection with various deletion plasmids. (C) IP analysis assessing the interaction between Smurf2 and CASC3 in HL-60 cells following transfection with different deletion plasmids. (D) Ubiquitination assay assessing Smurf2-mediated ubiquitination of CASC3 in HL-60 cells post-transfection with various deletion plasmids. (E) Western blot analysis of whole-cell lysates (WCLs) from HL-60 cells transfected with indicated plasmids. (F) Ubiquitination assay in HL-60 cells post-transfection with indicated plasmids.

CASC3 protein contains several domains, and, to determine those involved in Smurf2-mediated ubiquitination and degradation, we constructed several plasmids with deletions of various regions: 1–136, 1–351, 137–283, and 352–703 (Figure 4A). We found that deletion of either 1–351 or 137–283 blocked Smurf2-induced degradation of CASC3 in HL-60 cells (Figure 4B). To further explore this, IP analysis was conducted to determine whether these deletions affected the interaction between Smurf2 and CASC3 in HL-60 cells. We found that both deletions (1–351 and 137–283) remarkedly attenuated the interaction between Smurf2 and CASC3 in HL-60 cells (Figure 4C). Deletion of the 1–136 failed to block Smurf2-mediated degradation of CASC3 or their binding (Figures 4B and 4C), suggesting that the 137–283 domain is crucial for Smurf2-mediated degradation of CASC3. Moreover, ubiquitination assay showed that deletions of 1–351 or 137–283 conferred resistance to Smurf2-mediated ubiquitination of CASC3 (Figure 4D). Mutagenesis studies further revealed that mutation of lysine 254 to arginine (K254R) rendered CASC3 resistant to Smurf2-induced degradation, while mutations at K344R and K419R did not have this effect (Figures 4E and S5). Strikingly, the ubiquitylation assay confirmed that K254R mutation abolished Smurf2-mediated ubiquitylation of CASC3 (Figure 4F). Altogether, these findings suggest that the 137–283 domain and K254 could be critical for Smurf2-mediated ubiquitination and degradation of CASC3 in leukemia.

### Downregulation of Smurf2 promotes cell viability via CASC3

**Figure 5:**
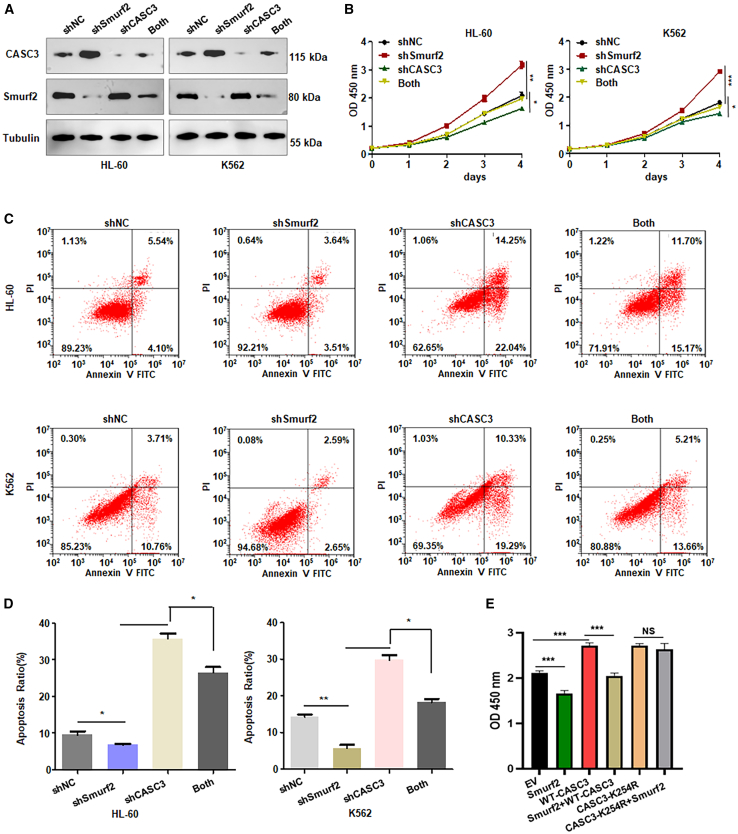
Downregulation of Smurf2 promotes leukemia cell viability via CASC3 (A) Western blot analysis of Smurf2 and CASC3 expression in HL-60 and K562 cells post-shSmurf2 and shCASC3 infections. (B) CCK-8 assay assessing cell viability in HL-60 and K562 cells post-shSmurf2 and shCASC3 infections. (C) Cell apoptotic death analysis in HL-60 and K562 cells post-shSmurf2 and shCASC3 infections. (D) Quantitative data for (C). ∗*p* < 0.05; ∗∗*p* < 0.01. (E) CCK-8 assay assessing cell viability in HL-60 cells with Smurf2 cDNA and CASC3 cDNA transfections. Data are representative of three independent experiments. The data are presented as the mean ± SD. *n* = 3. ∗*p* < 0.05; ∗∗*p* < 0.01; ∗∗∗*p* < 0.001. ns, no significance.

To explore whether the depletion of Smurf2 enhances cell viability through governing CASC3 expression in leukemia, we infected HL-60 and K562 cells with shSmurf2 and shRNA CASC3 (shCASC3). As expected, shCASC3 infection reduced the expression of CASC3 in both HL-60 and K562 cell lines (Figures 5A and S6). Additionally, shSmurf2-mediated increase in CASC3 expression was negated by shCASC3 infection in both leukemia cell lines (Figures 5A and S6). CCK-8 assays further demonstrated that shCASC3 infection inhibited cell viability in HL-60 and K562 cells, while shSmurf2 increased cell viability (Figure 5B). Consistently, the suppression of cell viability by shCASC3 was reversed by shSmurf2 infection in both HL-60 and K562 cells (Figure 5B). Flow cytometry data also revealed that shCASC3 infection induced early apoptosis from 4.1% to 22.04% and late apoptosis from 5.54% to 14.25% in HL-60 cells (Figure 5C). Similarly, in K562 cells, shCASC3 induced early apoptosis from 10.76% to 19.29% and late apoptosis from 3.71% to 10.33% (Figures 5C and 5D). In contrast, shSmurf2 infection inhibited early apoptosis from 10.76% to 2.65% in K562 cells (Figures 5C and 5D). Notably, shCASC3-induced apoptosis was effectively abrogated by shSmurf2 infection in both HL-60 and K562 cells (Figures 5C and 5D). Moreover, CASC3 overexpression abrogated Smurf2 upregulation-inhibited cell viability in HL-60 cells (Figure 5E). Thus, Smurf2 knockdown promotes cell viability in part via regulating the expression of CASC3 in leukemia cells.

### Overexpression of Smurf2 retards tumor growth in mice

**Figure 6:**
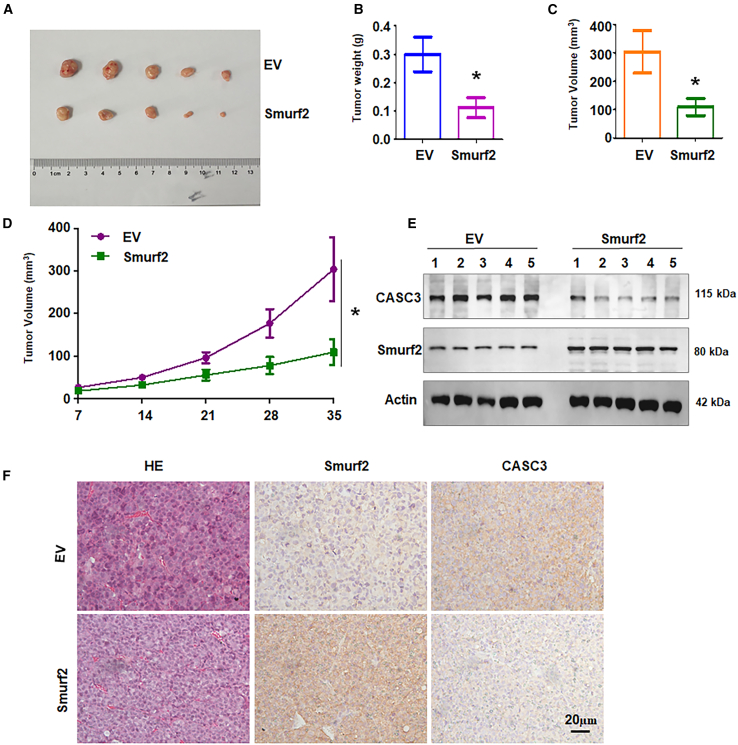
Overexpression of Smurf2 retards tumor growth in mice (A) Representation images of resected tumors from nude mice injected with HL-60 cells stably overexpressing Smurf2 after 5 weeks. (B) Representative images of tumor masses in mice at 5 weeks. (C) Tumor volumes recorded in mice over 35 days. (D) Tumor volume progression illustrated weekly. (E) Western blot analysis of Smurf2 and CASC3 expression in tumor tissues. (F) Immunohistochemistry staining for Smurf2 and CASC3 in tumor tissues. Data are representative of three independent experiments. The data are presented as the mean ± SD. *n* = 5. ∗*p* < 0.05.

To further validate the function of Smurf2 *in vivo*, we performed experiments using a mouse model. HL-60 and K562 cells with stable Smurf2 overexpression or CASC3 knockdown were subcutaneously inoculated into the flanks of nude mice. After 5 weeks, the tumors from mice with Smurf2 overexpression exhibited significantly reduced growth compared to control mice (Figures 6A, 6B, and S7A). Tumor volume data further confirmed that overexpression of Smurf2 led to reduced tumor size in mice (Figures 6C, 6D, and S7A). Moreover, CASC3 knockdown reduced tumor growth in mice (Figures S7B and S7C). Western blotting analysis of tumor tissues showed that overexpression of Smurf2 reduced CASC3 expression (Figures 6E and S8). Immunohistochemistry staining of tumor sections also supported the finding that CASC3 expression was downregulated in tumors with Smurf2 overexpression (Figure 6F). Collectively, these results suggest that Smurf2 suppresses tumor growth *in vivo*, potentially by downregulating CASC3.

## Discussion

In the current study, we demonstrated that Smurf2 inhibits cell viability and triggers cell apoptosis in leukemia cells, identifying CASC3 as a potential substrate of Smurf2. Moreover, Smurf2 exerts its biological effects by regulation of CASC3 in leukemia. Several studies have suggested a role for Smurf2 in leukemia cells. For example, melanoma differentiation-associated gene-7/interleukin-24 was shown to suppress cell growth and colony formation in K562 cells by upregulation of p21 and BRCA2 and CDKN1A interacting protein (BCCIP) and downregulation of CDK6, Smurf2, and phosphorylated retinoblastoma (RB).^31^ Additionally, the bone morphogenic protein pathway, which includes Smurf2, Snail1, Smad7, ACV1C, and INHBA, has been implicated in cell-cycle progression and self-renewal in CML cells.^32^ In B-ALL cells, upregulation of Smurf2 was found to regulate HDAC3 and inactivate the Janus kinase/signal transducer and activator of transcription 3 (JAK/STAT3) pathway, leading to promotion of cell apoptosis.^33^ A chimeric protein, IL2-Smurf2, induced cell death in a mouse model of leukemia and lymphocyte.^34^ Smurf2 has also been shown to induce ubiquitination and degradation of YY1, leading to inhibition of B cell proliferation and lymphomagenesis.^35^ Consistent with these findings, we observed that Smurf2 attenuated cell viability in leukemia.

CASC3 has also been implicated in various cancers. Genomic analysis revealed that CASC3 is highly expressed in breast cancer with HER2/TOP2A co-amplification compared with HER2-amplified breast cancer.^36^ In gastric cancer patients, CASC3 depletion was associated with worse prognosis.^37^ Xu et al. reported that methylation-regulated miR-124-1 attenuated tumorigenesis by inhibiting CASC3 expression, leading to the inactivation of the c-Jun N-terminal kinase (JNK), ERK, and p38-mitogen-activated protein kinase pathways in hepatocellular carcinoma (HCC).^38^ Upregulation of CASC3 facilitated cell proliferation and colony formation in HCC cells, while depletion of CASC3 displayed the opposed phenotypes.^38^ Liu et al. further showed that CASC3 expression is upregulated in HCC tissues and cells, and its downregulation led to inhibition of cell migration, invasion, proliferation, and glycolysis in HCC cells. Moreover, circ_0091579 increased the expression of CASC3 via sponging miR-490-5p in HCC cells.^39^ In breast cancer, CASC3 expression was positively associated with patient survival.^40^ Inhibition of CASC3 suppressed the expression of circ-NOL10 in breast cancer. Overexpression of circ-NOL10 inhibited cell proliferation, migration, and invasion and induced apoptosis in breast cancer. This study suggested that CASC3 can regulate the expression of circ-NOL10 and govern the tumorigenesis and progression of breast cancer.^41^ In line with these findings, our study shows that knockdown of CASC3 inhibits cell viability and induces apoptosis in leukemia cells, suggesting that CASC3 plays a potential oncogenic role in leukemia.

Previous studies have used proteomic methods and discovered that BLZF1 and CASC3 are substrates targeted by tankyrase and RNF146 E3 ligase for degradation.^42^ In our work, we identified that CASC3 is a substrate of Smurf2 in leukemia cells. Strikingly, the 137–283 domain of CASC3 plays a critical role in Smurf2-mediated ubiquitination and degradation of CASC3 in leukemia. In conclusion, the Smurf2/CASC3 axis may act as a potential therapeutic target for leukemia therapy.

### Limitations of the study

Despite identifying CASC3 as a new substrate of Smurf2, several limitations remain in our study. It is essential to determine whether an association between Smurf2 and CASC3 exists in leukemia patient samples. Moreover, further studies should assess whether Smurf2 overexpression inhibits tumor growth through regulation of CASC3 *in vivo*. Although this study utilized cell lines and mouse models, further validation using conditional Smurf2 knockout mice is warranted. Given that Smurf2 targets multiple substrates involved in tumorigenesis, it remains to be determined whether additional critical Smurf2 substrates contribute to leukemia development. Targeting CASC3 expression or enhancing Smurf2 levels may represent a promising therapeutic strategy. Notably, the natural compound schisandrin B has been reported to upregulate Smurf2 protein levels in colorectal cancer,^43^ while curcumin induces NLRP3-dependent pyroptosis in non-small cell lung cancer (NSCLC) (ncells by inhibiting Smurf2 ubiquitin ligase activity.^44^ Hence, it is important to investigate whether schisandrin B and curcumin can modulate Smurf2 expression in leukemia. Furthermore, the regulatory mechanisms controlling Smurf2 expression and its subsequent effect on CASC3 protein levels in leukemia remain unclear. This study does not address the downstream signaling pathways through which CASC3 promotes leukemia cell viability. Precious reports have identified RNF146 as an E3 ligase that regulates CASC3.^42^ However, whether Smurf2 functions redundantly or synergistically with RNF146 ligase in leukemia has yet to be determined. In conclusion, targeting the Smurf2/CASC3 axis may offer a potential therapeutic strategy for the treatment of leukemia.

## Resource availability

### Lead contact

Further information and requests for resources should be directed to the lead contract, Weijia Wang (wangwj@zsph.com).

### Materials availability

This study did not generate new unique reagents.

### Data and code availability

• This paper analyses existing, publicly available data. These accession numbers for the datasets are listed in the key resources table.
• This study does not report original code.
• Any additional information required to reanalyze the data reported in this paper is available from the lead contact upon request.

## Acknowledgments

This work was supported by Key Projects of 10.13039/501100007162Guangdong Provincial Department of Science and Technology (no. 2022B1515230007), 10.13039/501100003453Natural Science Foundation of Guangdong Province (no. 2021A1515011320), the Preservation and Innovative Development of Traditional Chinese Medicine (no. K2024B1002), and Zhongshan Key Research and Development Program (no. 2021B3001).

## Author contributions

R.Z. and J.L. performed the experiments, analyzed the data, and wrote the manuscript. F.L., M.H., T.L., B.C., Y.P., and Y.T. conducted the experiments and analyzed the data. P.W., J.W., and W.W. edited the manuscript and supervised the study. All authors approved the final revision.

## Declaration of interests

The authors declare no competing interests.

## STAR★Methods

### Key resources table

**Table:** 

REAGENT or RESOURCE	SOURCE	IDENTIFIER
**Antibodies**		

Anti-Smurf2	Cell Signaling Technology	Cat No. #12024
Anti-Tubulin	Cell Signaling Technology	Cat No. #2146
Anti-Myc	Proteintech	Cat No. 16286-1-AP
Anti-Flag	Sigma	Cat No. F1804
Anti-actin	Cell Signaling Technology	Cat No. #4967
Anti-CASC3	Abcam	Cat No. Ab90651
Anti-Bcl-2	Cell Signaling Technology	Cat No. #3498

**Chemicals, peptides, and recombinant proteins**		

RPMI-1640 medium	Gibco	Cat No. 11875119
Cell Counting Kit-8	Beyotime	Cat No. C0041
Annexin V-FITC Apoptosis Detection Kit	Sigma	Cat No. CBA059
cycloheximide	Sigma	Cat No. 66-81-9
Trizol	Invitrogen	Cat No. 15596018
cDNA Synthesis SuperMix Kit	TransGen	Cat No. AT341-01
SYBR Green PCR kit	Qiagen	Cat No. 204054
Lipofectamine 2000	Invitrogen	Cat No. 11-668-019

**Experimental models: Cell lines**		

HL-60	ATCC	Cat No. CCL-240
U937	ATCC	Cat No. CRL-1593.2TM
K562	ATCC	Cat No. CCL-243

**Experimental models: Organisms/Strains**		

BALB/c-nu/nu mice	SLAC	Cat No. Mice

**Software and algorithms**		

GraphPad Prism	GraphPad	Version 7.0
Flowjo	BD Biosciences	Version 10
ImageJ	NIH	https://imagej.nih.gov/ij/

### Experimental model and study participant details

#### Cell lines and cell culture

The AML cell lines HL-60 and U937 cells, and CML cell line K562, were bought from ATCC (Manassas, VA, USA). HL-60 and K562 cells were cultured in RPMI-1640 medium (Invitrogen, Carlsbad, CA, USA) in a humidified environment at 37°C with 5% CO_2_. Short Tandem Repeat (STR) analysis were used for cell line authentication. All cell lines were tested for mycoplasma contamination. The culture medium was supplemented with 10% fetal bovine serum (FBS; Invitrogen), 100 U/ml of penicillin, and 100 U/ml of streptomycin.

#### Animal experiments

BALB/c-nu/nu mice (*n* = 10, male, aged 3–5 weeks) were purchased from SLAC Co. Ltd (Shanghai, China) and randomly allocated into two groups (5 mice per group). HL-60 and K562 cells (1 × 10^7^), with Smurf2 overexpression or CASC3 depletion, were mixed with Matrigel in 100 μL of PBS and injected subcutaneously into the flanks of the nude mice. Tumor sizes were measured weekly using a digital caliper, and tumor volume was calculated using the formula V = A × B^2^ × 0.52, where A is the longest diameter and B is the shortest diameter. At specified time points, tumors were excised, weighed, and analyzed 35 days post-injection. All animal experiments were approved by the Institutional Animal Care and Use Committee of Zhongshan People’s Hospital (2022B1515230007) and conducted in compliance with ARRIVE guidelines. Excised tumors were subjected to Western blotting and immunohistochemistry to measure the expression of Smurf2 and CASC3.

### Method details

#### Cell transfection

Leukemia cells were grown in 6-well plates and, once they reached 60–70% confluence, were transfected with various indicated plasmids using Lipofectamine 2000 (Invitrogen, USA). shControl, 5′-TAC AAA CGC TCT CAT CGA CAA G-3′; shSMURF2#1, 5′-AGC GAG ACC TGG TTC AGA A-3′; shSMURF2#2, 5′-TGG AAG AAT CCA GTA TCT A-3′; shCASC3, 5′-CTG ATG ACA TCA AAC CTC GAA GAA T-3′. shRNAs targeting the open reading frames of Smurf2 or CASC3 were synthesized by GenePharma (Shanghai, China). HEK293T cells were co-transfected with packaging plasmids (pSPAX2 and pMD2.G) and either shSmurf2 or shCASC3 constructs. After 48 h, lentiviral supernatants were collected, filtered through a 0.45 μm membrane, and used to infect leukemia cells in the presence of 10 μg/mL Polybrene (GenePharma, Shanghai, China). Infected cells were selected with 2 μg/mL puromycin for several days. After infection, the cells were cultured for the indicated durations, as described in the results section.

#### Quantitative real-time PCR (RT-PCR)

Trizol reagent (Invitrogen) was applied to extract total RNA from leukemia cells. Reverse transcription was carried out with the cDNA Synthesis SuperMix Kit (TransGen, Beijing, China). RT-qPCR was performed using the SYBR Green PCR kit (Qiagen, USA). The relative mRNA expression levels were calculated using the 2^−ΔΔCt^ method and normalized to β-actin. The PCR primers used were as follows: Smurf2 forward 5′-ATC CTC GGC TGT CTG CTA ACT TG-3′; reverse 5′- CGA TACC ACT TGC TGT TGC TGT TG-3′; CASC3 forward 5′-CGC CAA GAG TGC TGA GGA GTC-3′; reverse 5′- TTC ACC TTC TTC ACC TTC CGA GTC-3′; β-actin forward 5′-GGA GAT TAC TGC CCT GGC TCC TA-3′; reverse 5′-GAC TCA TCG TAC TCC TGC TTG CTG-3′.

#### Western blotting

Cells were lysed in RIPA buffer (Beyotime, Shanghai, China), and total protein concentration was measured using a BCA protein assay kit (Pierce, USA). The proteins were then separated via SDS-PAGE and transferred onto PVDF membranes (Millipore, Billerica, MA, USA). The membranes were blocked with 5% non-fat milk for 1 h and subsequently incubated overnight at 4°C with primary antibodies. The primary antibodies included anti-Smurf2 (#12024, Cell Signaling Technology), anti-Myc (16286-1-AP, Proteintech), anti-Flag (F1804, Sigma), anti-Tubulin (#2146, Cell Signaling Technology), anti-CASC3 (ab90651, Abcam company), anti-actin (#4967, Cell Signaling Technology), and anti-Bcl-2 (#3498, Cell Signaling Technology) antibodies. Afterward, the membranes were incubated with secondary antibodies, and protein detection was conducted using the ECL chemiluminescent system (Thermo Fisher Scientific, Rochester, NY, USA). Protein levels were quantified using a Bio-Rad Imaging System.^45^

#### Cell viability assay

The Cell Counting Kit-8 (CCK-8) assay was used to evaluate the viability of leukemia cells. Transfected cells (5000 cells/well) were plated in 96-well plates and cultured in RPMI-1640 medium for the specified time points. Subsequently, 10 μL of CCK8 reagent was added to each well, and the cells were incubated for 4 h at 37°C. The absorbance was measured at 450 nm using a microplate reader (Bio-Rad, Hercules, CA, USA).

#### Cell cycle assay

Transfected leukemia cells were seeded into 6-well plates. After 48 h, the cells were harvested and fixed in 70% cold ethanol overnight at 4°C. Following fixation, the cells were washed with PBS, resuspended in PBS containing 0.1 mg/mL RNase I and 50 mg/mL propidium iodide (PI), and incubated at 37°C in the dark for 30 min. Cell cycle distribution was analyzed using a FACS flow cytometer (BD Biosciences, San Jose, CA, USA).

#### Apoptosis assay

Apoptosis was assessed using the Annexin V-FITC/PI Apoptosis Detection Kit (Sigma, St. Louis, MO, USA). Leukemia cells were cultured in 6-well plates and collected after 48 h. The cells were washed three times with PBS, resuspended in 500 μL of binding buffer, and stained with PI reagent and Annexin V-FITC (fluorescein isothiocyanate) for 20 min in the dark at room temperature. Apoptotic cells were subsequently identified by a FACS flow cytometer (BD Biosciences, San Jose, CA, USA) as described before.^46^

#### Immunoprecipitation (IP)

Transfected cells were rinsed thrice with PBS and lysed using immunoprecipitation lysis buffer, while vortexing intermittently on ice for 30 min. Cell debris was then eliminated by centrifugation at 12,000 rpm for 20 min and protein concentrations were determined with a BCA assay. 1 mg of lysate was incubated overnight with antibody-conjugated beads in a cold room. After incubation, the beads were washed three times with the immunoprecipitation buffer, resuspended in buffer. The bound proteins were separated by SDS-PAGE, followed by Western blotting for further analysis.^47^

#### Liquid chromatography-tandem mass spectrometry (LC-MS/MS)

HEK293T cells were transiently transfected with Flag-Fbxo2 and lysed 48 h later in IP buffer containing protease and phosphatase inhibitors. Lysates were immunoprecipitated using anti-Flag M2 affinity gel, and the Fbxo2 complexes were washed and eluted under denaturing conditions. Eluates were resolved by SDS-PAGE, and gel bands were excised, subjected to trypsin diagestion, and the resulting peptides were extracted, desalted, and analyzed by LC-MS/MS using a high-resolution mass spectrometer.

#### Cycloheximide chase assay

A cycloheximide (CHX) chase assay was performed to assess the stability of Smurf2 and CASC3 proteins in leukemia cells. Transfected cells were treated with 100 μg/mL cycloheximide for varying time intervals. Subsequently, cells were collected, lysed, and analyzed by Western blot analysis to evaluate protein levels at the indicated time points as previously described.^48^

#### Ubiquitination assay

HL-60 cells were transfected with plasmids encoding Flag-Smurf2, Myc-CASC3, and His-Ub, respectively. After 24 h, the cells were treated with 10 μM MG132 for 10 h. Cells were collected, washed three times with PBS, and lysed in ubiquitination assay buffer. The lysates were incubated overnight with an anti-Ub antibody 4°C. Immunocomplexes were then pulled down with Protein A/G plus agarose beads overnight at 4°C. Western blot analysis was performed to determine ubiquitinated CASC3 as described previously.^49^

#### Immunohistochemistry (IHC)

Tissue samples were fixed in 4% paraformaldehyde, embedded in paraffin, and sectioned using a microtome. Sections were incubated with primary antibodies, followed by HRP-conjugated secondary antibodies, and subsequently stained with DAB and counterstained with hematoxylin. IHC images were obtained by an optical microscope, and signal quantification was performed with ImageJ software.

### Quantification and statistical analysis

Statistical analyses were conducted using GraphPad Prism 7, with data presented as means ± S.D. Statistical significance between two groups was determined using a two-tailed independent Student’s t test, while multiple group comparisons were made using one-way ANOVA. A P-value of less than 0.05 was considered statistically significant.

## Supplemental information

Document S1. Figures S1–S8

Data S1. Original Western blotting images
