# Supplementary material for: Smurf2 enhances ubiquitin-mediated degradation of CASC3 and attenuates leukemia progression
Source: iScience. 2025 Aug 21;28(9):113411. doi: 10.1016/j.isci.2025.113411 (PMC12446198; doi:10.1016/j.isci.2025.113411)
Supplement: Document S1. Figures S1–S8 [file mmc1.pdf]

**iScience, Volume *28***

## Supplemental information

# Smurf2 enhances ubiquitin-mediated degradation of CASC3 and attenuates leukemia progression

**Ronghao Zeng, Jing Liu, Fen Lu, Ming Hong, Ting Lan, Baijian Chen, Yunping Pu, Yuwei Tan, Peter Wang, Juan Wang, and Weijia Wang**

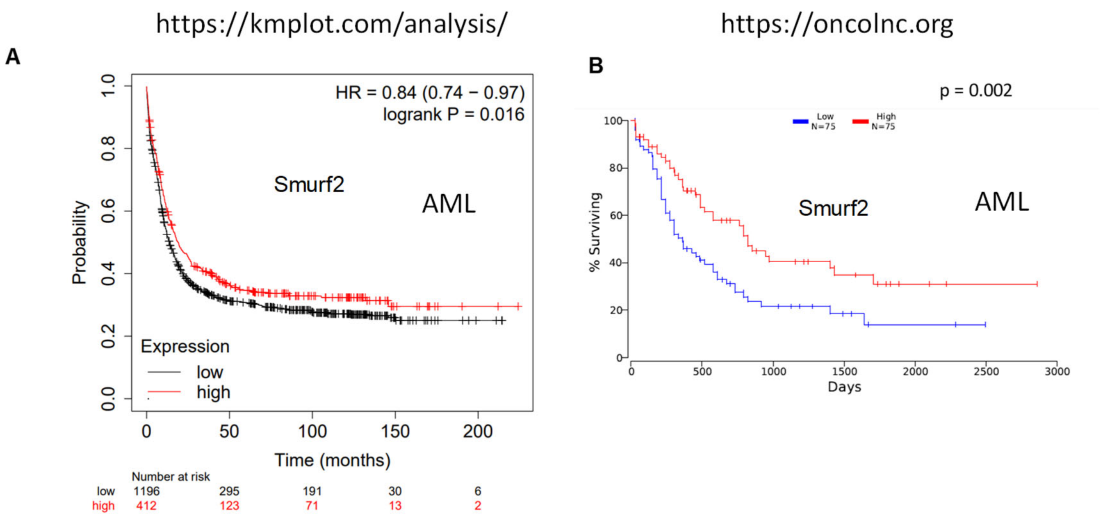


Supplementary figure 1: AML patients with high expression of Smurf2 displayed better survival outcomes. A.) https://kmplot.com was used. B.) https://oncolnc.org was used.

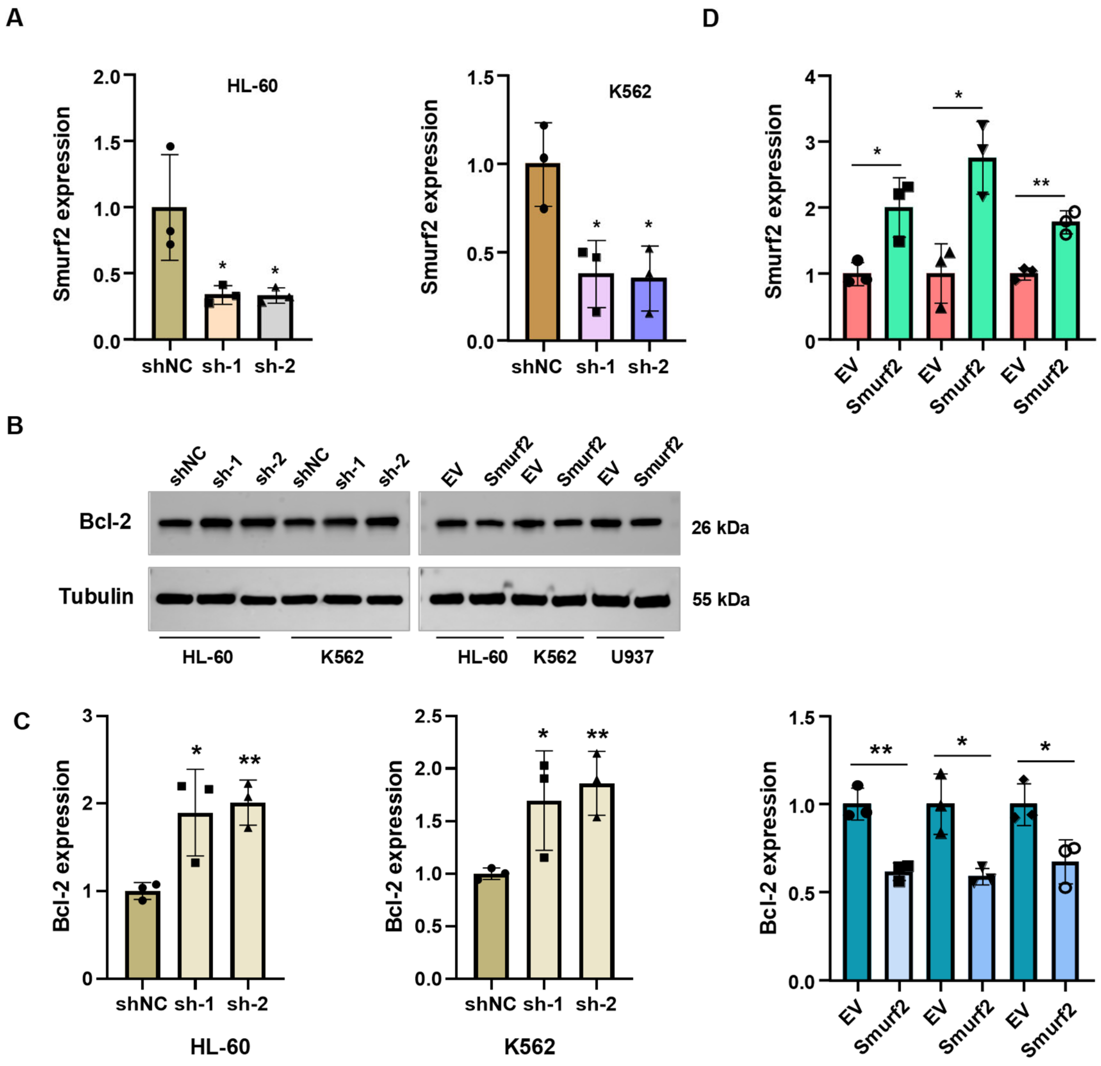


Supplementary figure 2: Bcl-2 expression was detected in HL-60 and K562 cells after Smurf2 modulation. A.) Quantitative data for Fig 1A are illustrated. B.) Western blotting analysis of Bcl-2 in HL-60, K562, and U937 cells after Smurf2 modulations. C.) Quantitative data for panel B are illustrated. D.) Quantitative data for Fig 2A are illustrated. Data are representative of three independent experiments. The data are presented as the mean ± S.D. *P < 0.05; **P < 0.01.

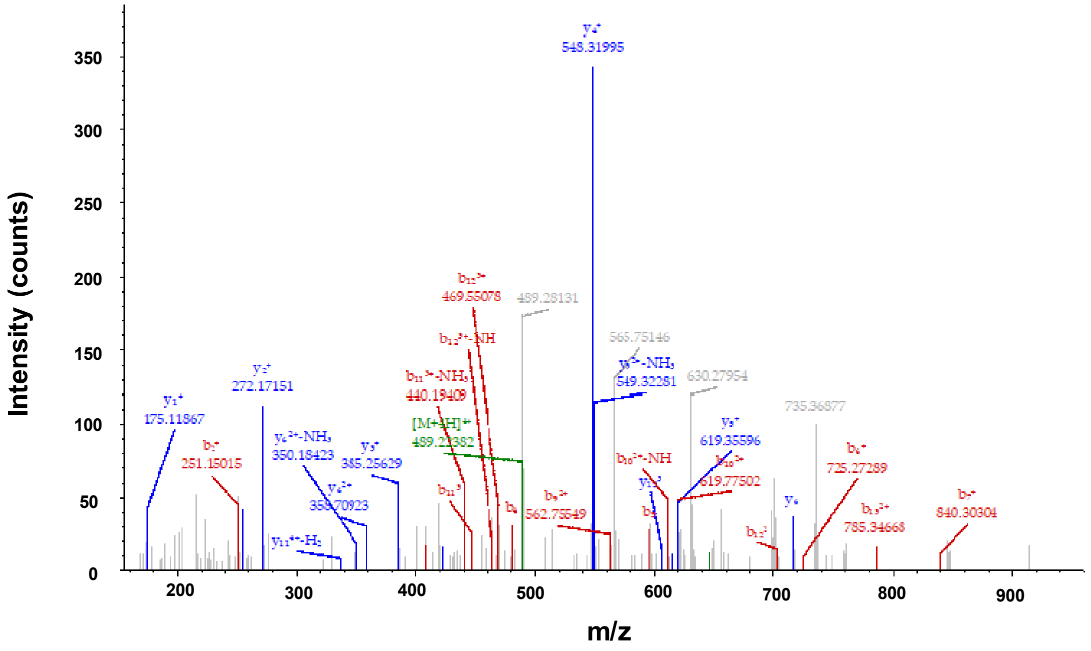


Supplementary figure 3: A mass spectrometry analysis was performed in HL-60 cells after Smurf2 plasmid transfection. The result identified CASC3 as a potential Smurf2-interacting protein.

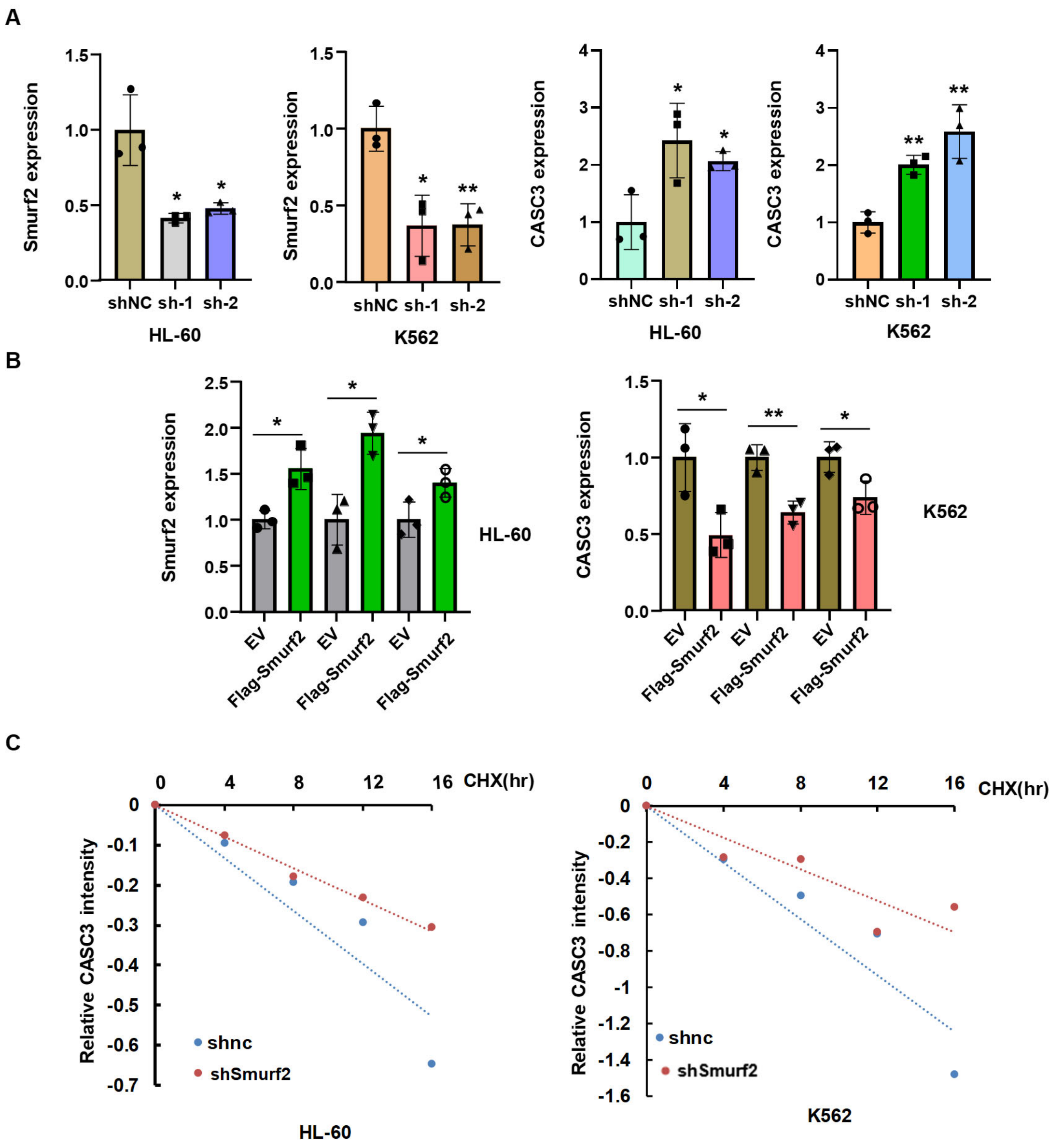


Supplementary figure 4: Smurf2 regulates CASC3 expression and its half-life. A, Quantitative data for Fig. 3B are illustrated. B, Quantitative data for Fig. 3C are illustrated. C. Quantitative data for Fig. 3G are illustrated. Data are representative of three independent experiments. The data are presented as the mean ± S.D. *P < 0.05; **P < 0.01.

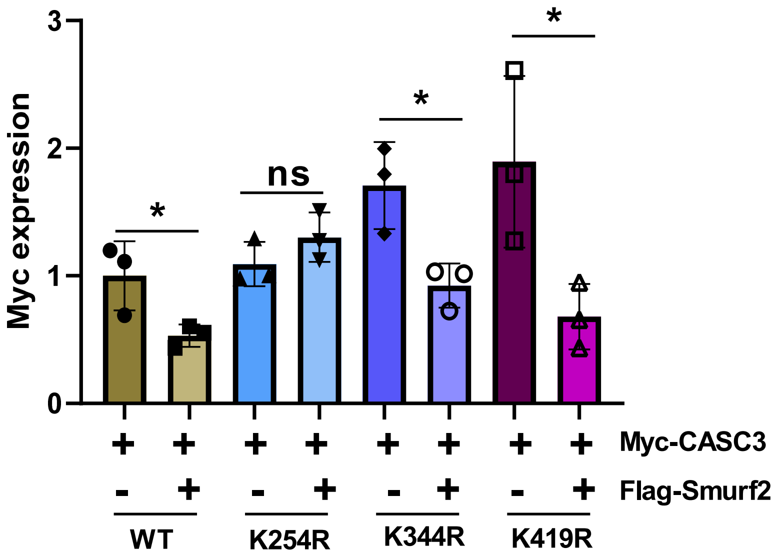


Supplementary figure 5: Quantitative data for Fig. 4E are illustrated. Data are representative of three independent experiments. The data are presented as the mean ± S.D. *P < 0.05; ns: no significance.

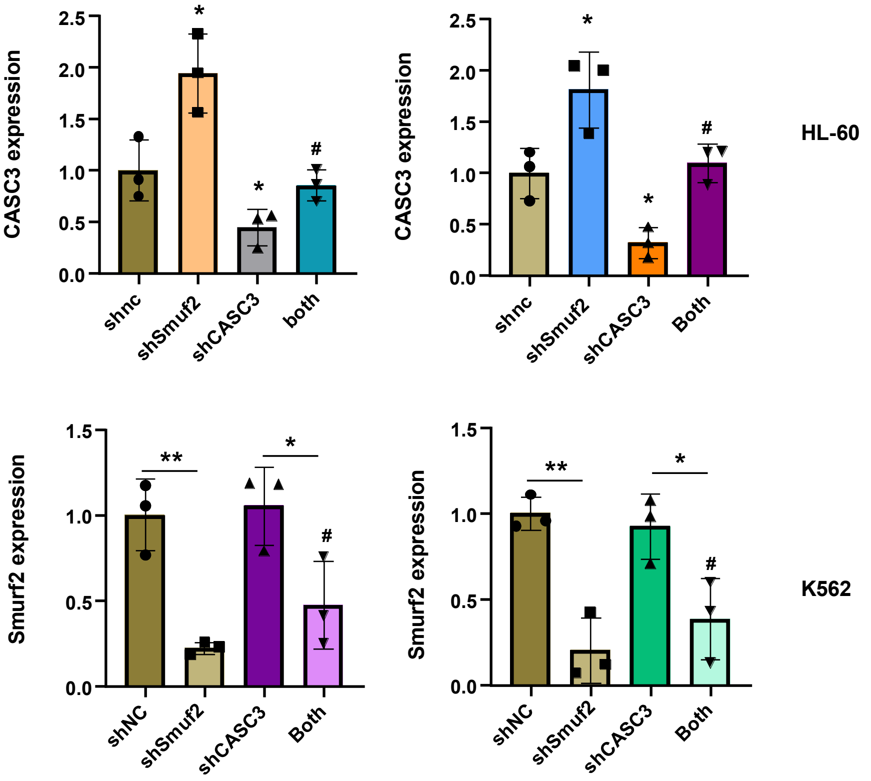


Supplementary figure 6: Quantitative data for Fig. 5A are illustrated. Data are representative of three independent experiments. The data are presented as the mean ± S.D. *P < 0.05; **P < 0.01; #: compared with shSmurf2 or shCASC3 alone.

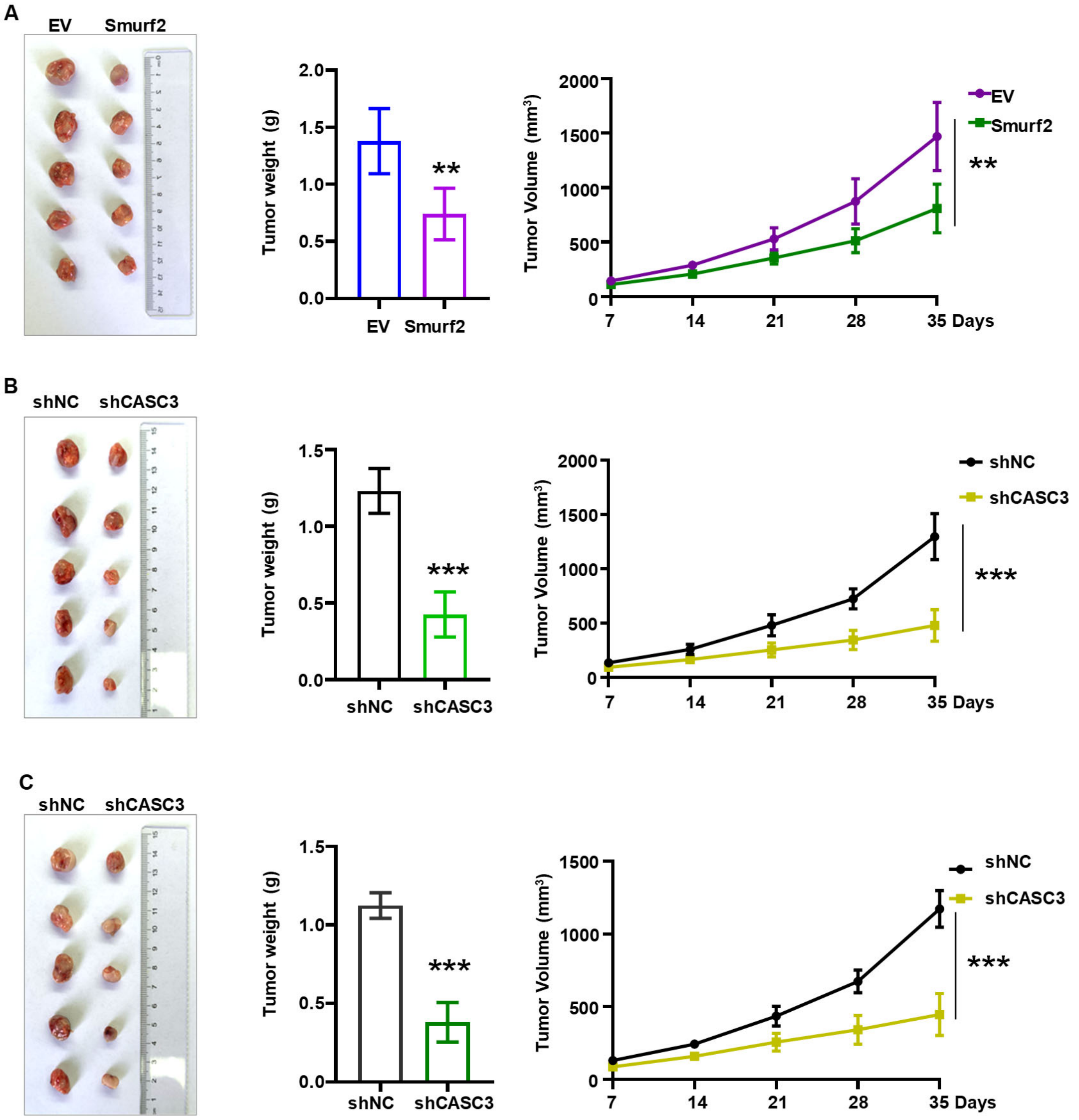


Supplementary figure 7: Smurf2 overexpression and CASC3 knockdown reduced tumor growth in mice. A: Representation images of resected tumors from nude mice injected with K562 cells stably overexpressing Smurf2 after 5 weeks (left panel). Representative images of tumor weight in mice at 5 weeks (middle panel). Tumor volume progression illustrated weekly (right panel). B: Representation images of resected tumors from nude mice injected with HL-60 cells with shCASC3 infection after 5 weeks (left panel). Representative images of tumor weight in mice at 5 weeks (middle panel). Tumor volume progression illustrated weekly (right panel). C: Representation images of resected tumors from nude mice injected with K562 cells with shCASC3 infection after 5 weeks (left panel). Representative images of tumor weight in mice at 5 weeks (middle panel). Tumor volume progression illustrated weekly (right panel). The data are presented as the mean ± S.D. n = 5. **P < 0.01; ***P < 0.001.

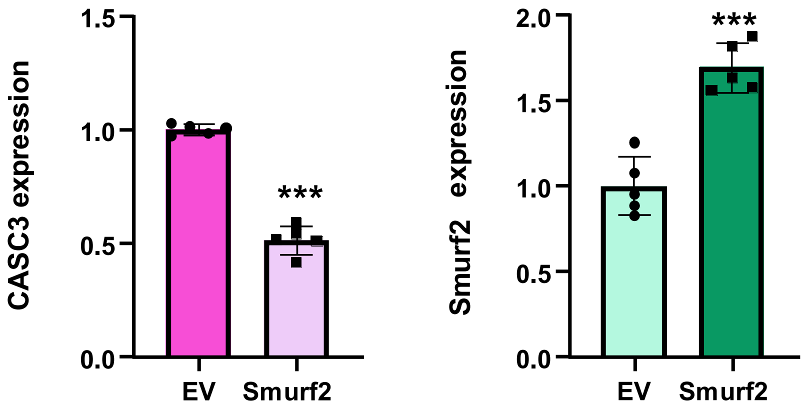


Supplementary figure 8: Quantitative data for Fig. 6E are illustrated. The data are presented as the mean ± S.D. n = 5, ***P < 0.001;
