# Supplementary material for: Smurf2 enhances ubiquitin-mediated degradation of CASC3 and attenuates leukemia progression
Source: iScience. 2025 Aug 21;28(9):113411. doi: 10.1016/j.isci.2025.113411 (PMC12446198; doi:10.1016/j.isci.2025.113411)
Supplement: Data S1. Original Western blotting images [file mmc2.pdf]

Fig 1 A

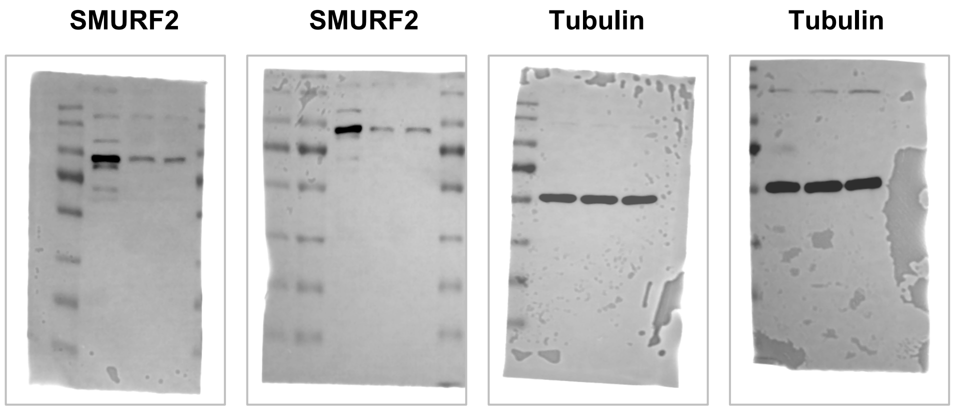

Fig 2 A

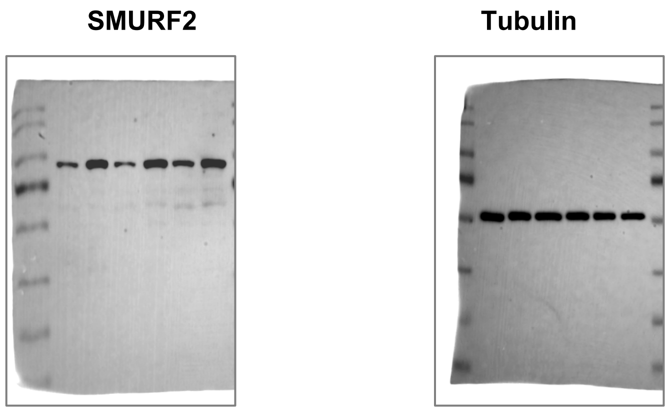

Fig 3B

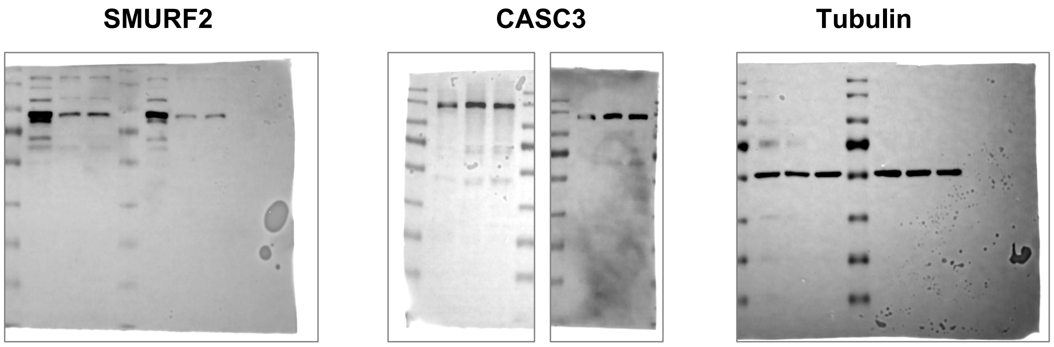

Fig 3C

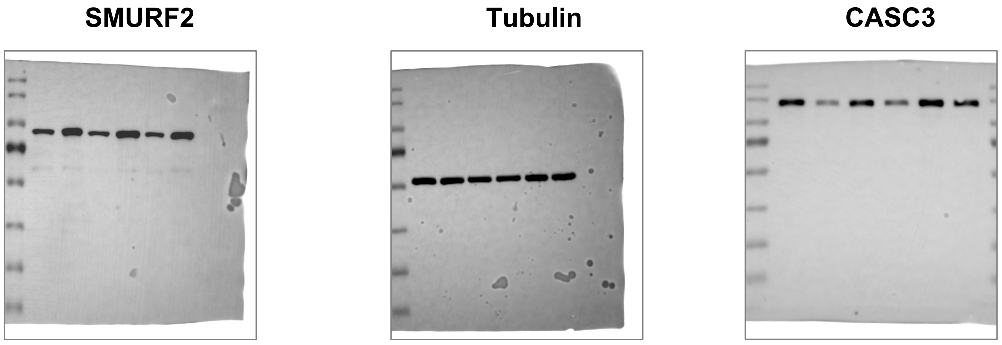

Fig 3D

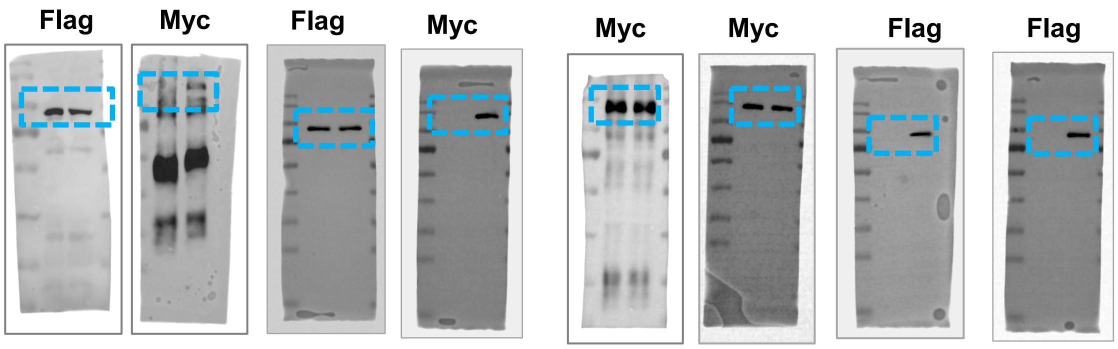

Fig 3E

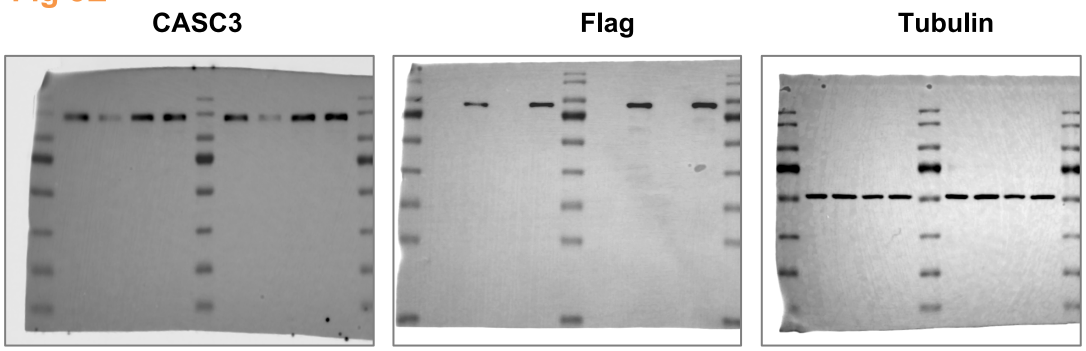

Fig 3F

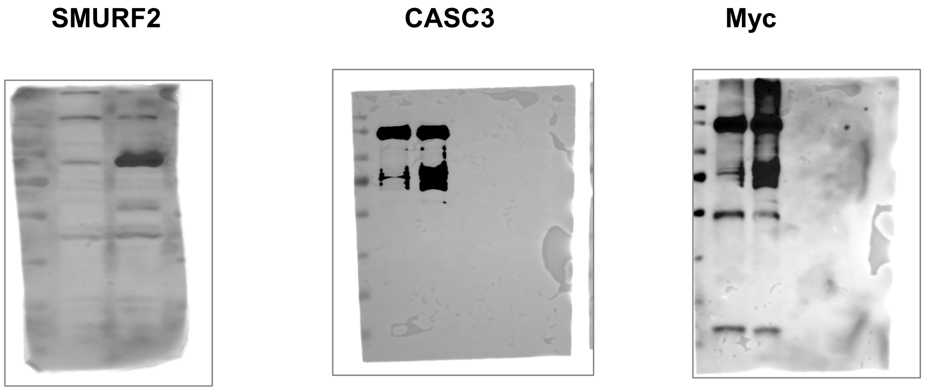

Fig 3G

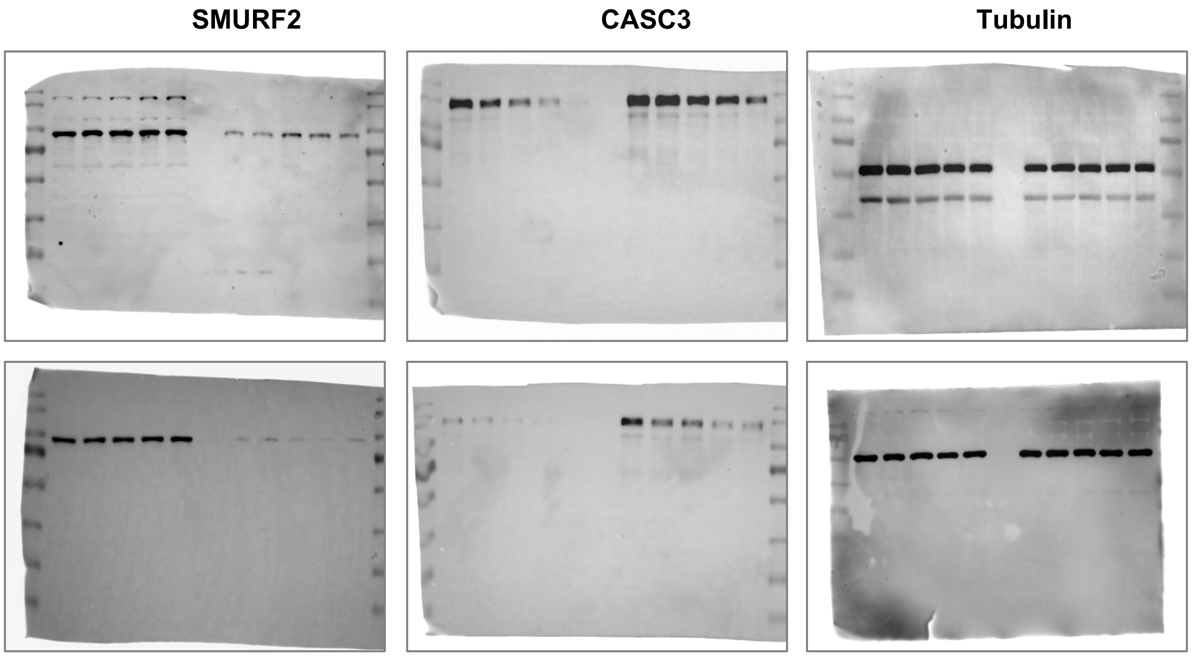

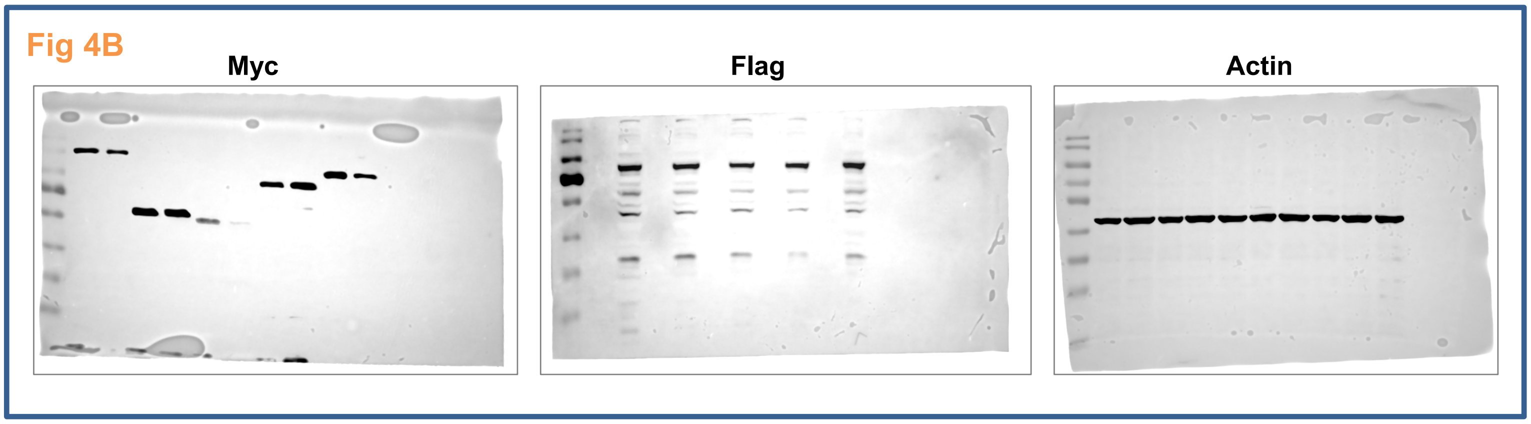
Fig 4B
Myc
Flag
Actin

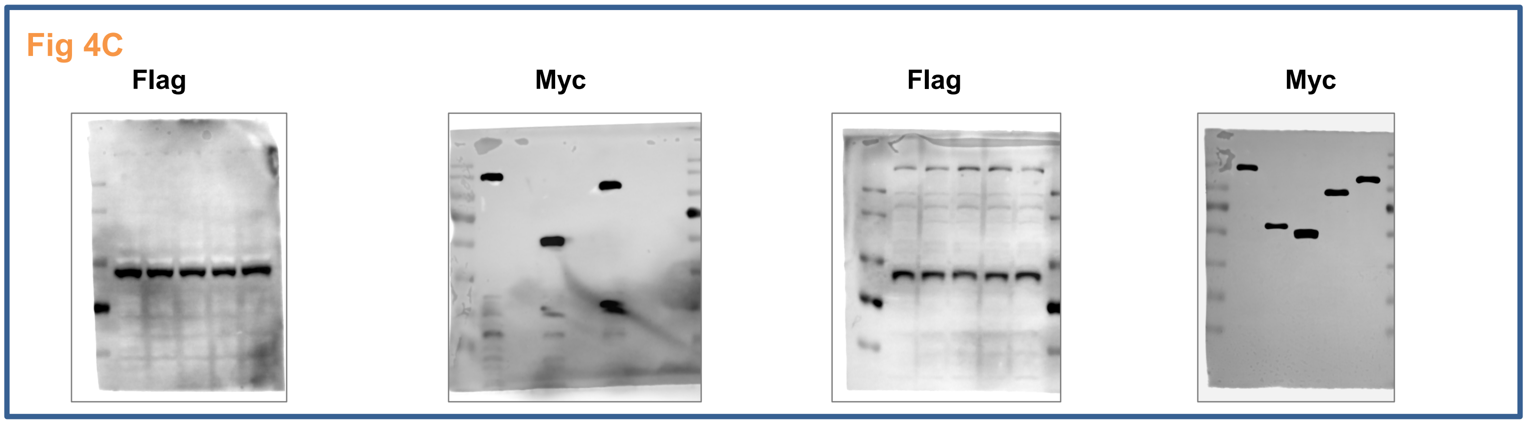
Fig 4C
Flag
Myc
Flag
Myc

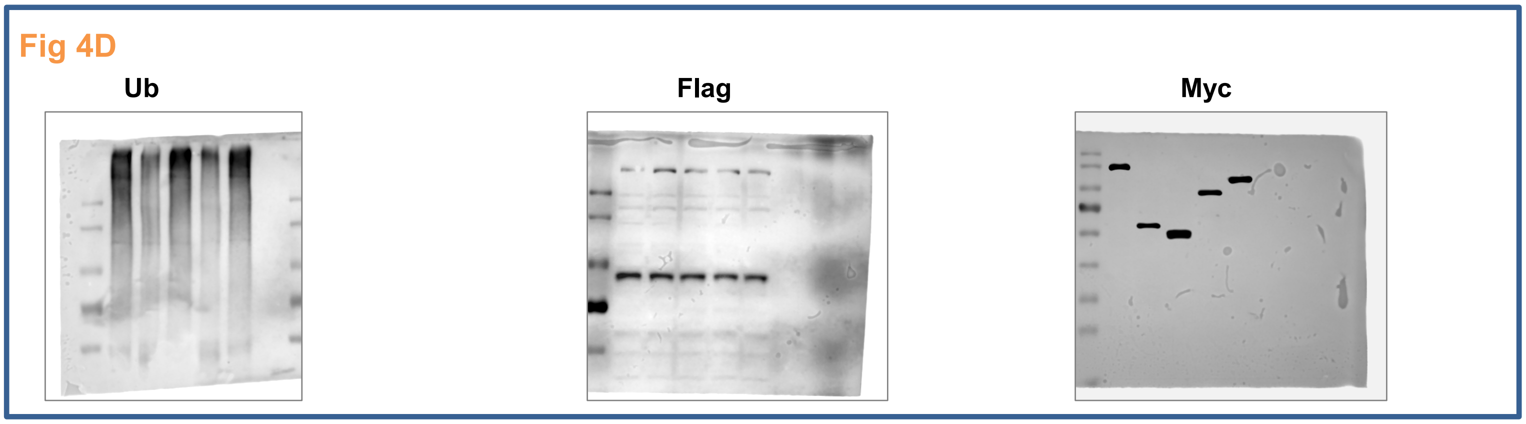
Fig 4D
Ub
Flag
Myc

Fig 4E

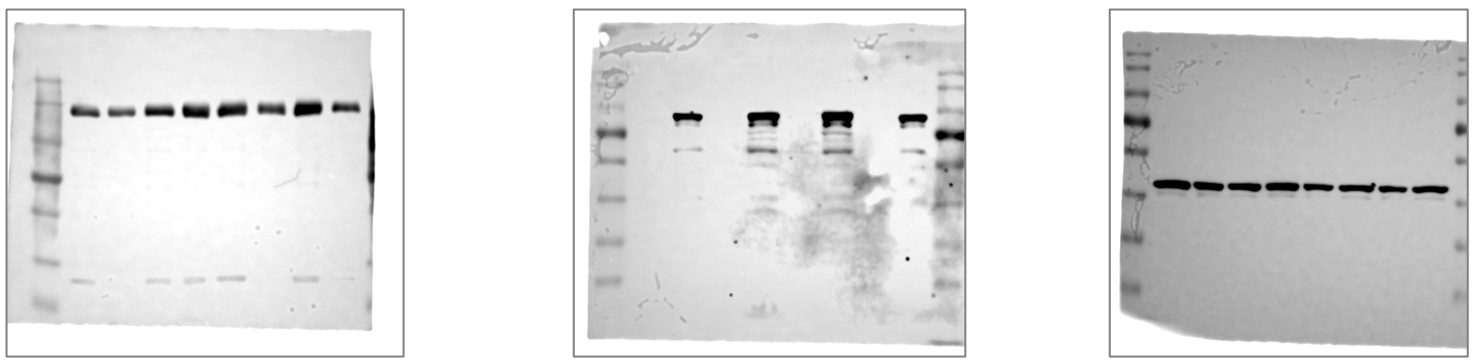

Fig 4F

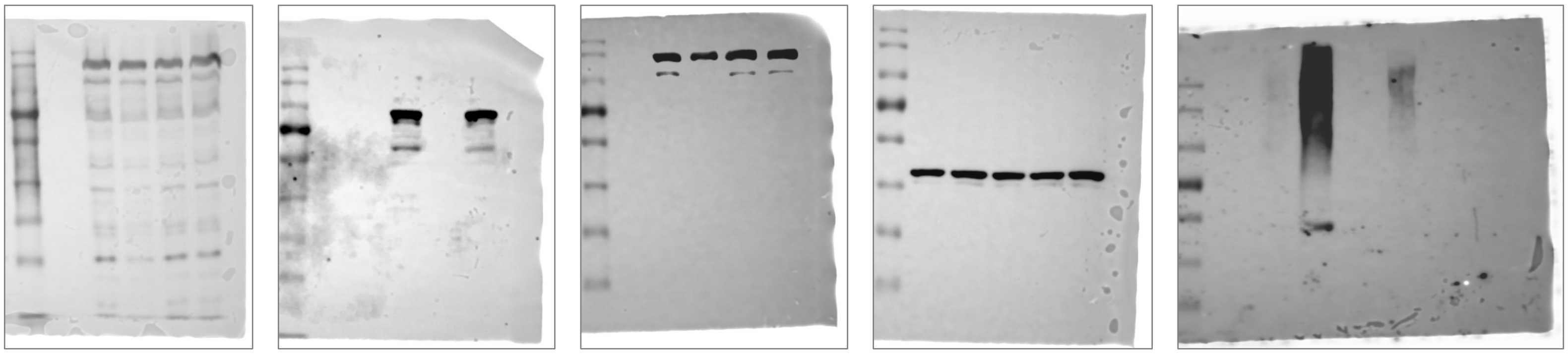

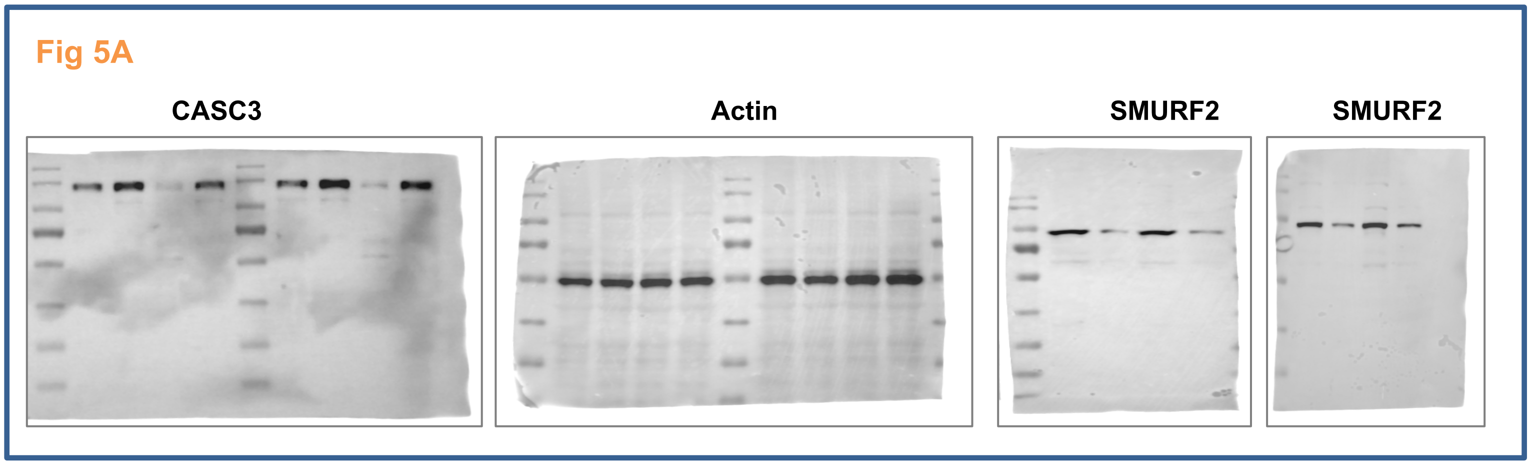

Fig 5A
CASC3
Actin
SMURF2
SMURF2

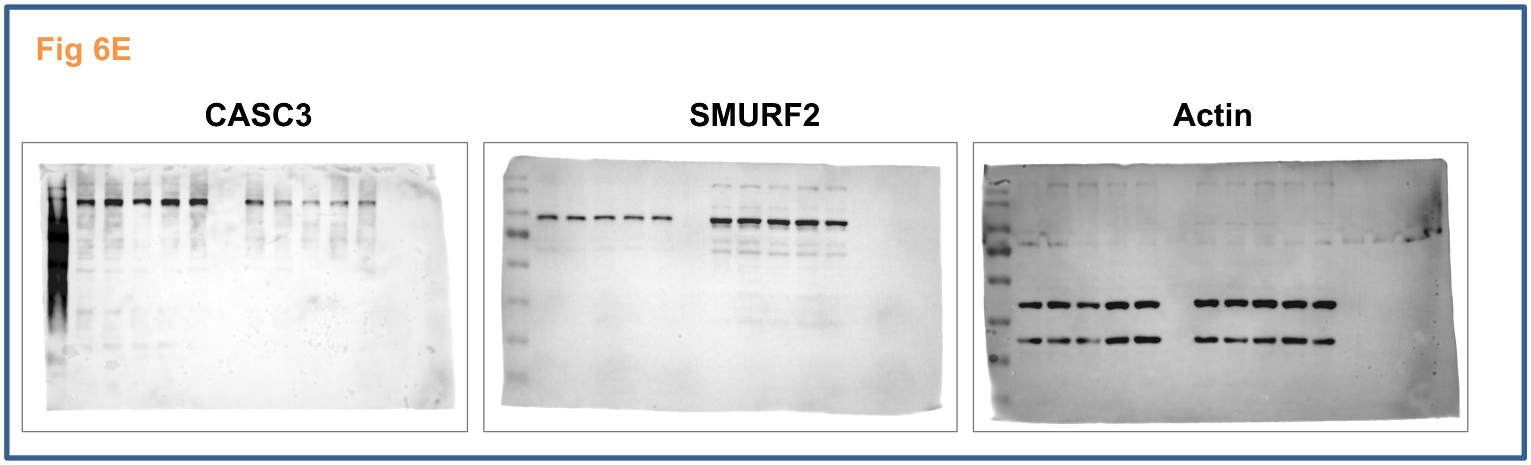

Fig 6E
CASC3
SMURF2
Actin

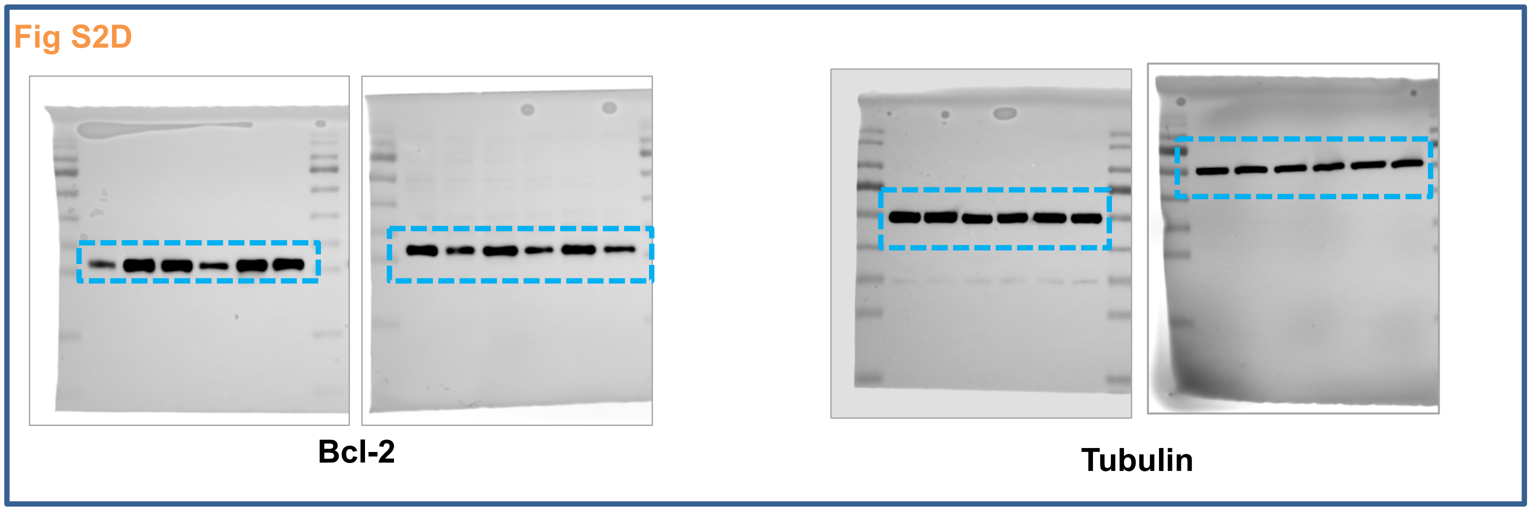

Fig S2D
Bcl-2
Tubulin
